# Neural representation of phonological wordform in temporal cortex

**DOI:** 10.3758/s13423-024-02511-6

**Published:** 2024-04-30

**Authors:** David O. Sorensen, Enes Avcu, Skyla Lynch, Seppo P. Ahlfors, David W. Gow

**Affiliations:** 1 Division of Medical Sciences, Harvard Medical School, Cambridge, MA USA; 2 Department of Neurology, Massachusetts General Hospital, Harvard Medical School, Boston, MA USA; 3 Athinoula A. Martinos Center for Biomedical Imaging, Massachusetts General Hospital, Charlestown, MA USA; 4 Department of Radiology, Massachusetts General Hospital, Harvard Medical School, Boston, MA USA; 5 Department of Psychology, Salem State University, Salem, MA USA; 6 Neurodynamics and Neural Decoding Group, Massachusetts General Hospital, 65 Landsdowne Street, rm 219, Cambridge, MA 02139 USA

**Keywords:** Phonology, Neural decoding, Neural representation, Spoken word, Recognition

## Abstract

While the neural bases of the earliest stages of speech categorization have been widely explored using neural decoding methods, there is still a lack of consensus on questions as basic as how wordforms are represented and in what way this word-level representation influences downstream processing in the brain. Isolating and localizing the neural representations of wordform is challenging because spoken words activate a variety of representations (e.g., segmental, semantic, articulatory) in addition to form-based representations. We addressed these challenges through a novel integrated neural decoding and effective connectivity design using region of interest (ROI)-based, source-reconstructed magnetoencephalography/electroencephalography (MEG/EEG) data collected during a lexical decision task. To identify wordform representations, we trained classifiers on words and nonwords from different phonological neighborhoods and then tested the classifiers' ability to discriminate between untrained target words that overlapped phonologically with the trained items. Training with word neighbors supported significantly better decoding than training with nonword neighbors in the period immediately following target presentation. Decoding regions included mostly right hemisphere regions in the posterior temporal lobe implicated in phonetic and lexical representation. Additionally, neighbors that aligned with target word beginnings (critical for word recognition) supported decoding, but equivalent phonological overlap with word codas did not, suggesting lexical mediation. Effective connectivity analyses showed a rich pattern of interaction between ROIs that support decoding based on training with lexical neighbors, especially driven by right posterior middle temporal gyrus. Collectively, these results evidence functional representation of wordforms in temporal lobes isolated from phonemic or semantic representations.

**Supplementary Information:**

The online version contains supplementary material available at 10.3758/s13423-024-02511-6.

## Introduction

Neural decoding analyses of activity in the posterior temporal gyri have provided important insight into the nature of neural sensitivity to segmental features in spoken language (Bhaya-Grossman & Chang, 2022; Mesgarani et al., 2014; Oganian & Chang, 2019; Yi et al., 2019). In contrast, it is less clear how neural activity reflects any aspects of specifically lexical knowledge (Poeppel & Idsardi, 2022). The goal of the present study is to isolate and localize neural representations of wordform – activity indexing sound patterns that differentiate individual words and mediate the mappings between acoustic-phonetic input and word-specific semantic, syntactic, and articulatory information.

Wordforms play a central role in lexically mediated language-dependent processes ranging from phonetic interpretation, word learning, lexical segmentation, and perceptual learning to sentence processing and rehearsal processes in working memory (Bresnan, 2001; Ganong, 1980; Gathercole et al., 1999; Merriman et al., 1989; Norris et al., 2003). However, questions as basic as how time is represented (Gwilliams et al., 2022; Hannagan et al., 2013), whether wordform representations are episodic or idealized (Pierrehumbert, 2016), morphologically decomposable or holistic (Pelletier, 2012), or fully specified versus underspecified (Lahiri & Marslen-Wilson, 1991) remain topics of vigorous debate. It is even unclear whether words and nonwords have overlapping representation above the segmental level. Nonword processing is strongly affected by form similarity to known words (Bailey & Hahn, 2001; Frisch et al., 2000; Gathercole, 1995). This could be a byproduct of word recognition processes, or a partial function of distributed word-level representations that can also capture overlapping nonword form patterns. Understanding the basis of these similarity effects would clarify the interpretation of results that depend on word-nonword contrasts.

Many studies have examined sensitivity to lexical wordform properties, including cohort size (Gaskell & Marslen-Wilson, 2002; Kocagoncu et al., 2017; Marslen-Wilson & Welsh, 1978; McClelland & Elman, 1986; Zhuang et al., 2014), phonological neighborhood density (Landauer & Streeter, 1973; Luce & Large, 2001; Luce & Pisoni, 1998; Peramunage et al., 2011), and lexical competitor environment (Prabhakaran et al., 2006). These lexical and sublexical effects are considered to play a crucial role in understanding the functional architecture of spoken word recognition and phonotactic constraints that shape wordform representation (Albright, 2009; Hayes & Wilson, 2008; Magnuson et al., 2003; Samuel & Pitt, 2003). An even larger literature has explored BOLD imaging contrasts between words and nonwords (see reviews by Binder et al., 2009; Davis & Gaskell, 2009). Several models (Gow, 2012; Hickok & Poeppel, 2007) have synthesized this research, hypothesizing that the bilateral posterior middle temporal gyrus and perhaps the bilateral supramarginal gyrus mediate the mapping between acoustic-phonetic and higher-level representations. While this work coarsely localizes the likely site containing such mediating wordform representations, it does not isolate individual wordforms, which would be an important first step towards characterizing their representation.

Isolating wordforms poses significant challenges. The phonological patterning of wordforms is confounded with the patterning of the phonemes that make up words, making it difficult to discriminate between lexical and segmental representation. This problem is compounded by evidence for the influence of lexical factors on phoneme processing and representation in the brain (Gow et al., 2021; Gow et al., 2008; Gwilliams et al., 2022; Leonard et al., 2016; Myers, 2007). Moreover, auditory words may evoke activation of semantic, articulatory, syntactic, and episodic representations in addition to stored representations of phonological wordform. Finally, evidence that both spoken words and phonotactically legal nonwords briefly activate multiple lexical candidates (Tanenhaus et al., 1995; Zhuang et al., 2014; Zwitserlood, 1989) suggests the need to separate the activation of a target word from that of its temporarily coactivated lexical candidates with overlapping phonology.

Neural decoding techniques provide powerful means for investigating the information content of neural signals (Haynes & Rees, 2006; Kriegeskorte & Diedrichsen, 2019; Kriegeskorte & Kievit, 2013). Several studies have used decoding to reconstruct latent information from human brain activity and to understand the relationship between neural representations and cognitive content (Anderson et al., 2019; Choi et al., 2021; Naselaris et al., 2009). The opportunities afforded by these methods have been tempered in part by a tendency to equate decodability with encoding or representation. Decoding analyses reveal the availability of information in neural activity to support a given classification but do not discriminate between latent information and functional representation – which must both capture contrast and influence downstream processing. In addition, functional representation should be localized in a neurally plausible brain region (e.g., an area independently identified as a lexical interface or wordform area). Following Dennett (1987) and Kriegeskorte and Diedrichsen (2019), we suggest that the concept of representation only becomes useful to theory if it can be demonstrated that a representation is plausibly localized and influences downstream processing. Several strategies have been proposed for demonstrating that putative neural representations affect downstream processing. Grootswagers et al. (2018) probed the relationship between neural activity and behavioral response time in tasks hypothesized to depend on those representations. While promising, this approach may be challenging to implement because it requires the identification of a task in which responses directly tap a specific representation, and reaction times are not significantly affected by post-representation metalinguistic processing demands. Goddard and colleagues (Goddard et al., 2016) used Granger causation analyses to explore downstream neural dependencies. Gow et al. (2023) used a variant of Goddard et al.’s strategy in which the same signals that were used to decode contrasting patterns of syllable repetition in individual brain regions using support vector machine analyses were shown to drive moment-by-moment decoding accuracy in other successful decoding regions using a Kalman filter implementation of Granger causation. The implicated regions were posterior temporal areas independently implicated in the processing of spoken words. Gow et al. (2023) suggested that these results demonstrate the propagation of representations of syllable repetitions.

In this study, we used a transfer-learning neural decoding and integrated effective connectivity approach, based on region of interest (ROI)-oriented source-reconstructed MEG/EEG activity, to identify and examine wordform representations. We isolated wordform representations by training classifiers to discriminate between activity evoked by sets of words and nonwords with overlapping phonology, for example*, pick* and *pid* (neighbors of *pig*) versus *tote* and *tobe* (neighbors of *toad*). We then tested the classifiers' ability to discriminate between untrained hub words, for example, *pig* versus *toad*. We reasoned that classifiers trained on activity prior to word recognition or nonword rejection would rely on the segmental overlap between neighbors and hub words, but that classification after word recognition would reflect similarity in global activation patterns associated with the consolidated representation of lexical neighbors. Because neighbors were defined by form similarity rather than semantic similarity, we reasoned that transfer performance would specifically depend on overlap in form representation between hub words and their neighbors. The inclusion of phonologically overlapping nonwords further allowed us to discriminate between lexically mediated classification (which should only occur for words) and sublexical influences (common to both words and nonwords). By comparing the alignment of overlap between neighbors and hub words (initial CV vs. final VC), we were further able to examine the role of word onsets and offsets in decoding. Word onsets play an out-sized role in spoken word recognition (see, e.g., Marslen-Wilson & Tyler, 1980), and so evidence of a decoding advantage for training sets that share an initial CV would be consistent with the claim that decoding taps lexical representation rather than simple phonological overlap. Finally, to determine whether decoded patterns had causal influences on downstream processing, we used the implementation of Granger causality analysis developed in Gow et al. (2023) to determine whether within-ROI activation patterns that support decoding in one area influenced decoding performance in downstream processing areas.

## Methods

### Participants

Twenty subjects (14 female) between the ages of 21 to 43 years (mean 29.5, SD = 7.1 years) participated. All were native speakers of American English and had no auditory, motor, or uncorrected visual impairments that could interfere with the task. Human participation was approved by the Human Subjects Review Board, and all procedures were conducted in compliance with the principles for ethical research established by the Declaration of Helsinki.

### Stimuli

The stimuli consisted of spoken CVC words and nonwords. To limit the potential influence of gross low-level acoustic properties on classification, only stop consonants were used (/b,d,g,p,t,k/). Six words (*pig, toad, cab, bike, dupe, gut*) were chosen as hub words. These were each used to define a set of phonological neighbors. For each hub, we created three word and three nonword neighbors by changing only one phoneme, with the position of the changed phoneme counterbalanced across the three positions. Consonant changes involved either the voice or place feature. Changed vowels were selected to be in close proximity to the hub vowel in F1/F2 space, but this requirement was applied less strictly in order to generate word and nonword tokens. The full set of words and nonwords is included in the Online Supplementary Material (OSM; Table S1). The stimuli were recorded by two speakers, one male and one female. All final consonants were fully released, which provided a cue to item offset. After normalizing the duration to 350 ms and the intensity to 70 dB SPL, a Praat script was used to make additional versions of each token by scaling formant values and mean F0 to create eight discriminable virtual talkers with different vocal tract sizes (Darwin et al., 2003). This allowed us to both introduce spectral variability within the training set and increase the power of the design.

### Procedure

Simultaneous MEG and EEG data were recorded while the subjects completed a lexical decision task using these stimuli. As the subjects listened to the recorded stimuli, the task was to determine whether or not the token they heard was a valid English word and signal their judgment via a left-handed keypress on a response pad. The subjects were asked to respond as accurately as possible. Stimuli were presented in 16 blocks, with two non-consecutive blocks assigned to each virtual talker. Within a block, each of the hub words was presented twice and each neighbor once, for a total of 48 trials. Trials were presented in a pseudo-randomized order, with the constraint that the second presentation of a hub word could not directly follow the first presentation. Stimulus presentation was controlled via PsychToolbox for Matlab (Kleiner et al., 2007).

The MEG/EEG data were collected using a Vectorview system (MEGIN, Finland) with 306 MEG channels, a 70-channel EEG cap, and vertical and horizontal electrooculograms. The EEG data were referenced to a nose electrode during the recording. All data were low-pass filtered at 330 Hz and sampled at 1,000 Hz. Prior to the testing, the locations of anatomical landmarks (nasion, and left and right preauricular points), four head-position indicator (HPI) coils, the EEG electrodes, and over 100 additional surface points on the scalp were digitized using a FASTRAK 3D digitizer (Polhemus, Colchester, VT). The head position with respect to the MEG sensor array was measured at the start of each block via the HPI coils and was tracked continuously during task performance. In a separate session, T1-weighted structural MRIs were collected from each subject on a 3T Siemens TIM Trio scanner using an MPRAGE sequence.

### Behavioral analysis

Behavioral accuracy was analyzed using the lme4 (Bates & Bolker, 2012) and lmerTest (Kuznetsova et al., 2017) packages in R (R Core Team, 2023) to perform a logistic mixed-effects analysis of the relationship between accuracy and lexical class (two levels: Words vs. Nonwords). We ran the full model with word condition as the reference level. Lexical class was treated as a fixed effect. We used random intercepts and slopes for lexical class by participants and random intercepts for lexical class by items. We reported the model estimation of the change in accuracy rate (in log odds) from the reference category for each fixed effect (b), standard error of the estimate (SE), Wald z test statistic (z), and the associated p values.

### MEG/EEG preprocessing and source reconstruction

MEG/EEG data were processed offline using MNE-C (Gramfort et al., 2014) via the Granger Processing Stream software (Gow & Caplan, 2012). Eyeblinks were identified manually, and a set of signal space projectors corresponding to eyeblinks and empty room noise were removed from the MEG data. Epochs from -100 to 1,000 ms time-locked to the onset of the auditory stimuli were extracted after low-pass filtering the data at 50 Hz. Epochs with high magnetometer (> 100 pT) or gradiometer (> 300 pT/cm) values were rejected. The remaining epochs were averaged across all trials with a correct response for each subject.

All decoding and effective connectivity analyses were based on MEG/EEG source estimates for a set of ROIs. Source estimation enables the interpretation of the results in terms of brain regions, and also allows connectivity analyses between regions, reducing confounds due to the spatial spread of signals over several MEG and EEG sensors (Schoffelen & Gross, 2009). Minimum Norm Estimates (MNEs) were calculated for each individual subject to reconstruct event-related electrical activity in the brain (Hämäläinen & Ilmoniemi, 1994). The MEG/EEG source space consisted of ~10,000 current dipoles located on the cortical surfaces reconstructed from the structural MRIs using Freesurfer (http://surfer.nmr.mgh.harvard.edu/). For the MEG/EEG forward model, a three-compartment (Leahy et al., 1998) boundary element model was used, with the skull and scalp boundaries obtained from the MRIs. The MRI and MEG/EEG data were co-registered using information from the digitizer and the HPI coils. The MNE inverse operator was constructed with free source orientation for the dipoles. Source estimates were obtained by multiplying the MEG/EEG sensor data with the inverse operator. The source estimates for each individual subject were then brought into a common space obtained by spherical morphing of the MRI data using Freesurfer (Fischl et al., 1999) and averaged to create the group average source reconstruction that was used to perform ROI generation. For the ROI source waveforms used in the decoding and effective connectivity analyses, we calculated noise-normalized MNE time courses for each ROI using dynamic statistical parametric mapping (dSPM).

**Fig. 1:**
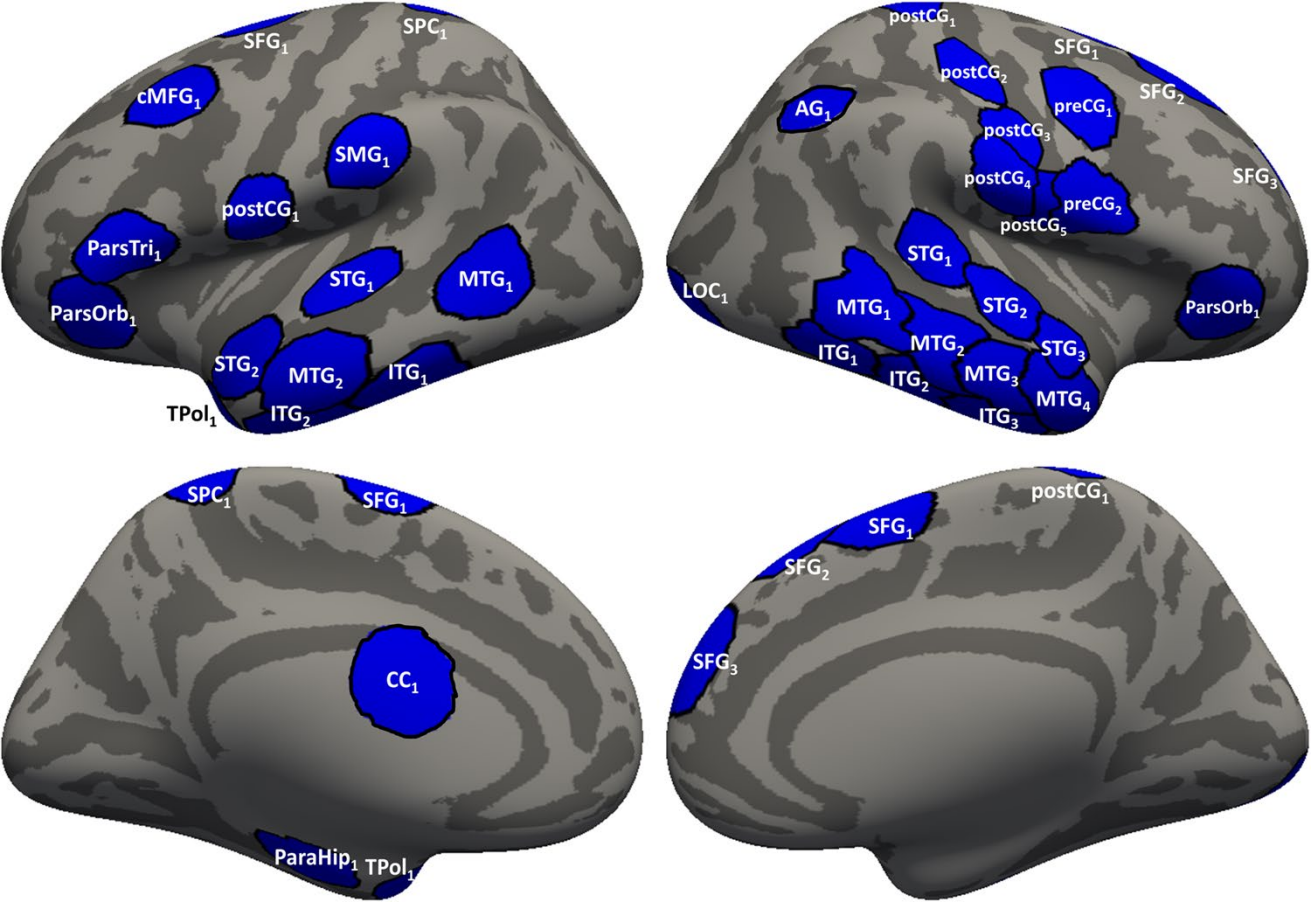
Regions of interest (ROIs) visualized over an inflated averaged cortical surface. Lateral (**top**) and medial (**bottom**) views of the left and right hemisphere are shown. ROI names are generated based on the location of the centroid vertex for each ROI in the Desikian-Killiany atlas parcellation of the average subject brain. For further description of the ROIs, see Table S2 (Online Supplementary Material)

ROIs were generated from the grand average evoked response using procedures previously described by Gow and Caplan (2012) designed to identify ROIs that meet the assumptions of Granger Causality analysis. Briefly, a set of potential centroid locations was generated consisting of the source space dipoles on the cortical surfaces with the highest activation in the time window from 100 to 500 ms after stimulus onset. From those centroids, neighboring dipoles were included into a growing ROI and distant dipoles were excluded from the final set of ROIs based on metrics of similarity, redundancy, and spatial weight. Because our decoding analyses rely on subdividing ROIs, it is beneficial to produce larger ROIs; we thus loosened our empirically determined similarity and redundancy constraints relative to previous studies (Gow & Nied (2014); see OSM Fig. 1 for an example ROI set created using previous parameters). This adjustment may increase the rate of type II errors through the inclusion of otherwise redundant signals but would not increase the likelihood of false-positive results in Granger analyses. This process resulted in a total of 39 ROIs, which were then transformed to each individual subject's source space.

For decoding analyses, individual ROIs were split into eight approximately equal-sized subdivisions. dSPM source time courses were calculated for each subdivision by averaging the source time courses of all the source dipoles within the subdivision. The mean value over the 100-ms pre-stimulus baseline period was subtracted, and the data were vector normalized across subdivisions for each timepoint in each epoch.

Source-space analyses of MEG and EEG significantly strengthen the interpretation of connectivity measures, compared with sensors-space analyses (Haufe et al., 2013). However, there will inevitably be some crosstalk between the estimated ROI source waveforms (Liu et al., 1998). Crosstalk can potentially lead to false-positive effects when a true effect in one ROI is falsely seen also in another ROI, or false-negative effects when the sensitivity to a true effect in one ROI is diminished due to the influence of crosstalk signal from another ROI without the effect. To minimize crosstalk effects, several steps were undertaken. First, we recorded simultaneous MEG and EEG, which can provide better spatial resolution than either method by itself (Sharon et al., 2007). Second, our data-driven algorithm for determining the ROIs was designed to maximize dissimilarity between the ROI source waveforms (Gow & Caplan, 2012). Third, effective connectivity analyses are based on prediction of future time points and thus less sensitive to the strictly spatial effects of crosstalk than zero-lag correlation-based connectivity measures (Nolte et al., 2004). Our previous studies of Granger causality among a relatively large number of ROIs (up to 68) have provided consistent results on speech and language-related processing (Avcu et al., 2023; Gow et al., 2008, 2023). Also, notably Michalareas et al. (2016) successfully analyzed effective connectivity among 26 human visual cortical areas using 275-channel MEG.

### Neural decoding

Neural decoding analyses with a transfer learning design were conducted to evaluate phonological neighborhood representations using support vector machine (SVM) classifiers (Beach et al., 2021). The SVM classifiers were trained to discriminate neighborhoods using only trials in which the neighbors were presented, then tested on their performance to discriminate the corresponding hub words in a transfer learning design. Pairwise classification was done at each time point within the epoch for all neighborhood pairs.

To increase the robustness of the ROI source waveforms as input to the SVM, bins of eight trials were randomly selected and averaged within each condition. The random bin assignment was repeated 100 times for each condition. Single timepoints from these bin averages were used as the input to the SVM, and the average classification accuracy across the 100 bin assignments was used as the measure of decoding accuracy for each time point. Performance of these classifiers was then averaged across all pairwise neighborhood contrasts. For statistical analysis, the decoding accuracy data were submitted to cluster-based permutation tests at the group level. Clusters were defined as consecutive time points of above chance performance (alpha = 0.05; chance performance = 50% for pairwise classification). The observed accuracy data for each subject were then randomly flipped with respect to chance accuracy (Beach et al., 2021) across 1,000 permutations, and the largest cluster within each permutation was taken to form a distribution of cluster sizes. The cluster statistic was Bonferroni-adjusted (for 39 ROIs) with *p* < 0.00128 needed to reach significance with a corrected alpha of 0.05.

Our primary decoding analyses contrasted the transfer discrimination of pairs of hub words based on training by exclusively word or nonword neighbors (Cheng et al., 2014). The purpose was to determine the degree to which decoding relied on stored representations of known words as opposed to sublexical overlap present in both neighboring words and nonwords. In addition, to further isolate the effects of sublexical overlap, we compared the decoding of hub word contrasts as a function of positional overlap between the neighbors and hub words. All nonwords in the study partially overlapped with real words, and any decoding based on nonword training could be due to this overlap. Given evidence for the relative importance of onsets in spoken word recognition (Marslen-Wilson & Tyler, 1980), we hypothesized that overlap between the initial CV- of words and nonwords in the training set and hub words (e.g., the neighbors *pick*, *pid*, and the hub word *pig*) would produce better decoding than overlap involving the final -VC of training words (e.g., *big*, *tig*, and *pig*). To compare between conditions, a second set of cluster permutation tests was performed with an additional constraint: timepoints had to both be above chance (uncorrected alpha < 0.05) and different than the comparator condition (uncorrected alpha < 0.05) to be included in clusters. Permutations randomly flipped the relationship of observed data with respect to both the chance accuracy and zero difference between conditions null values. Clusters with Bonferroni-adjusted (for 39 ROIs) *p* < 0.00128 were counted as significantly different between conditions (alpha = 0.05).

### Effective connectivity analyses

Effective connectivity analyses follow our previously published Granger causality analysis approach (Gow & Caplan, 2012), with modifications to integrate the results of the decoding analysis as described in Gow et al. (2023). The goal of the modified Granger causality analyses was to identify whether the activation time courses in ROIs that supported decoding could predict (or "Granger cause") the SVM classifier accuracy time courses in other ROIs. The integration involved substituting the single source waveform activation time course for each ROI (normally used in our Granger analyses) with data relevant to the decoding analyses. When evaluating the influence of a given ROI on others, the single activation time course for that ROI was substituted by the eight subdivision dSPM time courses used in the decoding analysis. When evaluating how other ROIs influenced a given ROI, the single activation time course for that ROI was substituted by the within-subject neural decoding accuracy time course averaged across all pairwise conditions from the decoding analysis based on word neighbors as the training set. All 39 ROIs were included in the predictive models, but only relationships between ROIs that showed significant transfer decoding for words were analyzed.

Representations, formally defined, require that the activity not only decode but also be related to a functional outcome (Dennett, 1987; Kriegeskorte & Diedrichsen, 2019). Our analysis thus focused on influences between regions that supported word-based transfer decoding to see how these activations reinforce each other when words were presented. Specifically, we examined the ability of the estimated activation time courses in a decoding ROI to predict the decoding accuracy in the other decoding ROIs. We selected the window of analysis to 250–550 ms, following latencies used for the lexically conditioned N400 ERP (Kutas & Federmeier, 2011). The strength of Granger causality, as quantified by the number of time points with a significant Granger Causality index, was compared in the single word condition to a control pair of ROIs (Milde et al., 2010) that did not exhibit transfer decoding in our analyses or have plausible processing relationship in this paradigm , L-cMFG1 and R-LOC1, using binomial tests.

## Results

### Behavioral results

Overall mean behavioral accuracy on the lexical decision was 90% (SD = 2.8%). Accuracy was higher for words (92%; *SD *= 4.4%) than for nonwords (86%; SD = 6.3%). This difference was statistically significant (*b* = 1.18, *SE* = 0.46, *z* =2.58, *p* =.01).

### Regions of interest

A set of 39 ROIs associated with overall task-related activation was identified through our process of grouping contiguous cortical source locations associated with activation peaks that share similar temporal activation patterns (Fig. 1, Table S2 (OSM)). These ROIs were used for neural decoding and effective connectivity analyses.

### Neural decoding: Lexicality effects

**Fig. 2:**
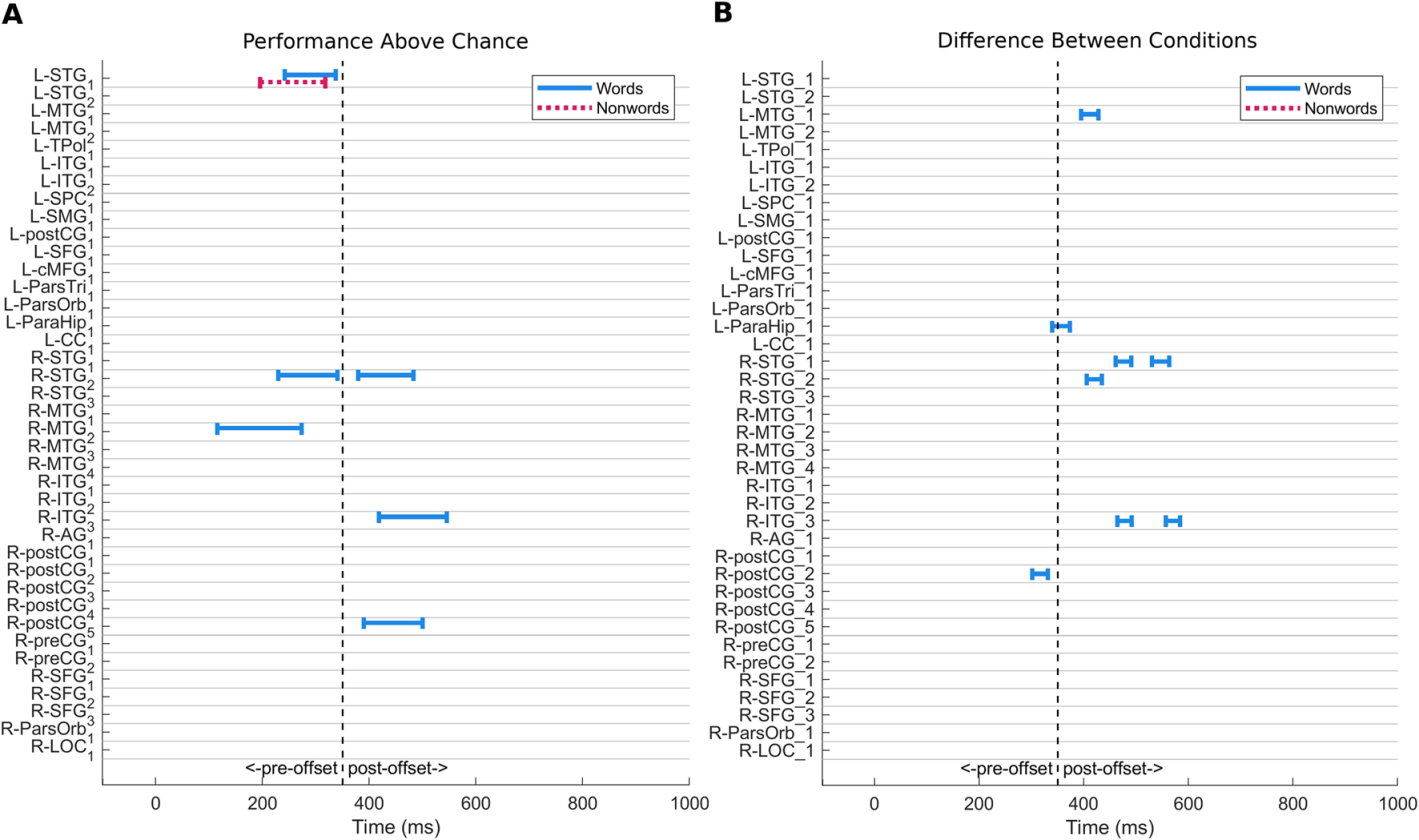
Bonferonni-corrected significant (corrected alpha = 0.05) transfer decoding clusters after training with word or nonword neighbors. (**A**) Clusters of significant above chance transfer decoding accuracy for words-only (blue solid) or nonwords-only (red dotted) conditions. (**B**) Clusters of transfer decoding accuracy which is above chance and significantly differs between training conditions. Better transfer decoding for words-only condition indicated by blue solid bars; no clusters of better transfer decoding for nonwords-only condition were observed. The vertical dotted line in both panels indicates the offset of the auditory stimuli

Figure 2 shows results of the transfer decoding analysis in which SVM classifiers were trained using exclusively either word or nonword neighbors and then tested on their ability to classify the corresponding hub words. Within the entire 1,100-ms epoch window, significant clusters of decoding time points were found in five of the 39 ROIs (Fig. 2A): L-STG_1_, R-STG_2_, R-MTG_2_, R-ITG_3_, and R-postCG_5_. With the exception of R-postCG_5_, which shows a brief earlier period of decoding, all decoding begins after the point (~ 225 ms) at which Gwilliams et al. (2022) identify the earliest decoding of the third phoneme of words in connected speech. All five of these produced successful transfer decoding of hub words when trained with word neighbors. When trained with nonword neighbors, only L-STG_1_ produced successful transfer decoding and only prior to stimulus offset. This timing falls after the potential decodability of the phoneme that makes training items nonwords, but possibly before all phonemic information is integrated in lexical representations. Overall, training with word neighbors resulted in better decoding across more ROIs than training with nonword neighbors.

Differences between conditions were observed in a total of six ROIs (Fig. 2B). All significant differences showed better decoding when trained with words than with nonwords. Prior to stimulus offset, two ROIs showed a difference between training conditions: L-ParaHip_1_ and R-postCG_2_. Four ROIs showed a difference during the post-offset period between 400 and 600 ms: L-MTG_1_, R-STG_1_, R-STG_2_, and R-ITG_3_. These differences are consistent with a lexically mediated effect.

### Neural decoding: Positional effects

We also examined the role of onset overlap between neighbors in the training set and hub word decoding. We hypothesized that neighbors that share a common CV onset with hub words would support more accurate hub word decoding than neighbors that share the same amount of overlap at their VC offsets. Within the entire epoch window, significant clusters of decoding time points were found in six of the 39 ROIs (Fig. S3A (OSM)) when classifiers were trained by neighbors that shared initial CV- sequences: L-STG_1_, R-STG_1_, R-STG_2_, R-STG_3_, R-postCG_5_ and R-preCG_2_. No ROIs produced successful transfer decoding when trained only by neighbors that shared final -VC sequences. Significant differences were found prior to stimulus offset for 13 ROIs (Fig. S3B (OSM)), with better decoding observed for initial CV- overlap in L-STG_1_, L-MTG_2_, L-ParsOrb_1_, R-STG_1_, R-STG_2_, R-STG_3_, R-MTG_2_, R-MTG_4_, R-ITG_1_, R-postCG_4_, R-postCG_5_, R-preCG_2_, and R-ParsOrb_1_. L-STG_1_ also supported better decoding for final -VC overlap over initial CV- overlap, but this was late in the epoch after the period typically associated with automatic spoken word recognition (Kutas & Federmeier, 2011). As predicted, we found better decoding was achieved using training neighbors with initial than with final overlap. Across both analyses, decoding supported by initial CV overlap generally occurred before stimulus offset. We believe this is due to the inclusion of nonwords in the training sets that failed to support lexical representation after they became inconsistent with lexical candidates.

### Effective connectivity analyses

**Fig. 3:**
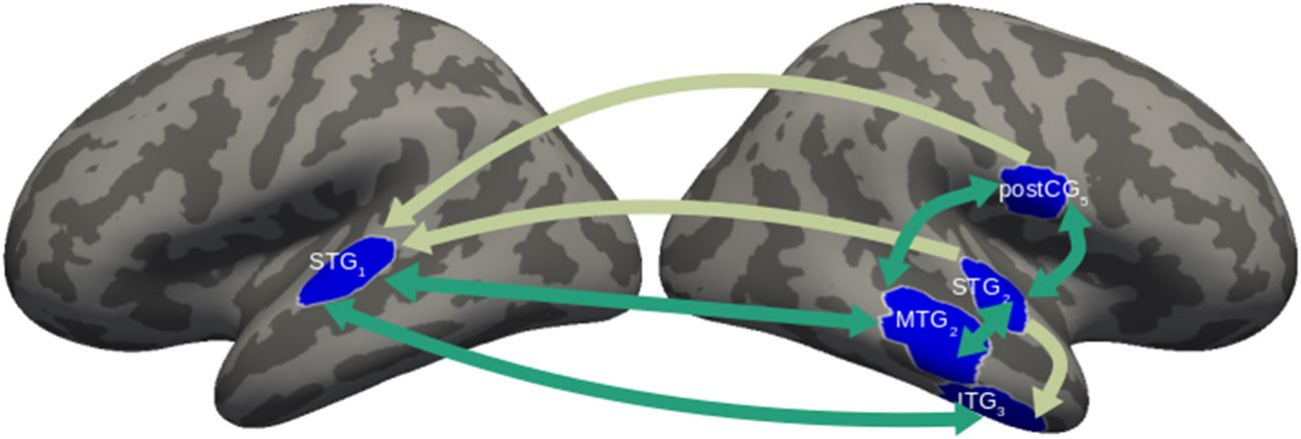
Effective connectivity analysis of transfer decoding regions of interest (ROIs). Effective connectivity between the five ROIs that supported reliable transfer decoding of hub words after training with word neighbors in the interval of 250–550 ms after the onset of the word. Lighter arrows indicate significant one-way directed Granger causation between ROIs. Darker green arrows indicate significant reciprocal connectivity. Significance is based on FDR-corrected binomial testing with an alpha of 0.05

To determine whether local patterns of neural activity influence downstream neural processing, we examined whether the event-related neural activity in any one of our decoding ROIs significantly influenced the decoding accuracy in the others within the 250- to 550-ms window associated with lexical processing indexed by the N400 component (Kutas & Federmeier, 2011). We focused on interactions among the five ROIs (L-STG_1_, R-STG_2_, R-MTG_2_, R-ITG_3_, and R-postCG_5_) that successfully discriminated hub words after training with real word neighbors of those hubs. FDR-corrected tests revealed 13 significant interactions (Fig. 3). These included reciprocal connections between R-MTG_2_ and L-STG_1_, R-STG_2_ and R-postCG_5_, as well as between L-STG_1_ and R-ITG_3_.We hypothesize that reciprocal relationships reflect a resonance dynamic that develops parity between representations, for example aligning wordform representations hypothesized to occur in posterior middle temporal gyrus/inferior temporal gyrus (MTG/ITG) (Gow, 2012; Hickok & Poeppel, 2007) with lower level segmental representations associated with superior temporal gyrus (STG) (Hickok & Poeppel, 2007; Mesgarani et al., 2014).

## Discussion

We undertook this study to isolate wordform representations and examine their neural basis. Wordforms were isolated from segmental overlap in time and from semantic overlap in feature space using phonological neighborhoods. We found significant decoding after training with either word or nonword neighbors prior to stimulus offset, consistent with incremental acoustic-phonetic processing before the presentation of the stimulus was complete. Indeed, when training was based on phonological overlap of the initial consonant, the same ROIs were able to decode, suggesting phonological overlap alone was sufficient to produce decoding in the word and/or nonword conditions early in the epoch. In contrast, post-stimulus offset decoding occurred mainly after training with word neighbors, suggesting lexical sensitivity in the wordform representation. This post-offset regime aligned with the well-characterized event-related potential evidence of lexical processing, including the N400 (Kutas & Federmeier, 2011). Additionally, phonological overlap alone did not produce transfer decoding in the immediate post-offset period, strengthening the case for lexical sensitivity. The lexically sensitive decoding occurred in a bilateral network of mostly temporal lobe regions. The effective connectivity analysis showed that the word-evoked activity in these regions preferentially influenced decoding accuracy in other decoding regions, with one region in right posterior middle temporal gyrus, R-MTG_2_, showing direct reciprocal influences on all but one of its fellow decoding regions (R-ITG_3_), and indirect influences on that area (mediated by interactions with R-STG_2_). Independent evidence that the bilateral posterior middle temporal gyrus is involved in lexical processing (see reviews by Gow, 2012, and Hickok & Poeppel, 2007) attests to the plausibility of this region as a site for wordform representation. To sum up, our results revealed a bilateral network of temporal lobe areas that were sensitive to both the phonology and lexicality of word-like stimuli and also affected downstream processing.

We achieved these results despite implementing a difficult decoding design. Rather than a leave-n-out cross-validation approach common to many decoding studies (Guggenmos et al., 2018; Hastie et al., 2009), we tested the classifiers in a transfer learning design with stimuli from multiple talkers. The SVMs were trained on one set of words or nonwords and tested on an untrained set of phonologically similar words, based on the assumption that phonologically similar words have overlapping patterns of distributed neural representation. Additionally, our ROI-based approach restricted the signals that the SVM could rely upon to classify epochs. We made these design choices to address qualities necessary to establish a representation. As formally defined by Dennett (1987) and Kriegeskorte and Diedrichsen (2019), for a signal to be a representation it must index the stimulus feature, affect downstream processing, and have a plausible localization. Our integrated approach allowed us to evaluate all three criteria: the decoding analyses tested for feature sensitivity; the effective connectivity analyses examined downstream effects; and the ROI-based approach addressed the location.

Most of the ROIs that decoded hub words based on training with words were in bilateral temporal lobe regions previously implicated a variety of spoken languages functions. Posterior middle temporal regions that decoded for words (combining results from the single condition and differences between condition analyses) include L-MTG_1_ and R-MTG_2_, located in regions that have been implicated in wordform representation. Posterior MTG and adjacent areas have been specifically implicated in wordform representations that mediate the mapping between sound and meaning (Gow, 2012; Hickok & Poeppel, 2007). Imaging studies have shown that activation in these posterior temporal regions is influenced by wordform properties such as word frequency, lexical neighborhood size, lexical enhancement/suppression, phonological similarity, and word-level structural properties (Biran & Friedmann, 2005; Gow et al., 2023; Graves et al., 2007; Prabhakaran et al., 2006; Righi et al., 2009). In addition, damage to posterior temporal regions has been shown to produce deficits in lexico-semantic processing (Axer et al., 2001; Coslett et al., 1987; Goldstein, 1948; Wernicke, 1970).

R-ITG_3_ is an anterior temporal ROI sensitive to wordform, located in a region that has been associated with word retrieval (Abrahams et al., 2003), attention to semantic relations (McDermott et al., 2003), and representing the semantic similarity among concepts (Patterson et al., 2007). Superior temporal ROIs included L-STG_1_ and R-STG_1,2,_, in regions that have been shown to be sensitive to the processing and representation of the sound structure of language (Hickok & Poeppel, 2007; Mesgarani et al., 2014). Effective connectivity studies of phonotactic repair in phoneme categorization judgments (Gow & Nied, 2014), phonological acceptability judgments (Avcu et al., 2023), and phonotactic frequency effects on lexical decision (Gow & Olson, 2015, 2016; Gow & Segawa, 2009; Gow et al., 2008) suggest the possibility of "referred lexical sensitivity" arising from resonance between acoustic-phonetic representation in superior temporal regions and lexical representation in middle temporal regions and supramarginal gyrus.

Outside of the temporal lobe, a ventral sensorimotor ROI, R-postCG_5_, showed decoding for words after stimulus presentation. This specific ROI aligns with a segment of the sensorimotor cortex involved in oral movements (Pardo et al., 1997), sensitive to the frequency of articulatory patterns (Treutler & Sörös, 2021) and associated with the perception of spoken language (Schomers & Pulvermüller, 2016; Tremblay & Small, 2011).

The positional analysis (Figs. S3 and S4 (OSM)) showed that word-initial overlap between training and test items supported decoding by more ROIs than did word-final overlap. This aligns with behavioral results that have shown that spoken word recognition relies more heavily on word onsets than offsets (Allopenna et al., 1998; Marslen-Wilson & Tyler, 1980; Marslen-Wilson, 1987). Despite this onset-bias, more ROIs decoded in the word analysis than in the onset analysis specifically in the post-offset period, suggesting that overlap at each position contributed to classification performance in the word condition. While phonological representations appear to persist for up to hundreds of milliseconds after presentation (Gwilliams et al., 2022), we did not observe transfer decoding based on phonological overlap after stimulus presentation. This implies that the neural activation patterns reflected parallel activation of stored words with overlapping phonology. Evidence for overlapping neural representation of phonologically similar words suggests a neural basis for lexically mediated "gang effects" supporting a variety of speech and spoken word recognition effects where less activated lexical candidates provide cumulative top-down support based on phonological overlap with input representations. This interpretation is consistent with the claims of the TRACE model (McClelland & Elman, 1986), in which cohort size, length of the target word, and phonetic saliency can modulate the intensity of gang effects and their role in onset effects, phonotactic repair, and categorical speech perception (Hannagan et al., 2013).

The results of our integrated effective connectivity analyses strengthen the argument for functional representation of decodable properties. The results show a dense pattern of interaction between decoding regions with activity that supports decoding of hub word contrasts based on training with their word neighbors influencing decoding accuracy in other ROIs. Significantly, most interactions are reciprocal and involve an ROI in lexically implicated right posterior middle temporal gyrus, R-MTG_2_ (Gow, 2012; Hickok & Poeppel, 2007). The reciprocal effective connectivity between this region and bilateral posterior STG (L-STG_1_ and R-STG_2_) is consistent with prior findings showing that behavioral evidence for lexical influences on speech perception coincides with increased Granger influences by posterior MTG on posterior STG (Gow & Nied, 2014; Gow & Olson; Gow & Olson, 2015; Gow et al., 2021). Whereas damage to posterior MTG is associated with lexical deficits, damage to posterior STG is not associated with specifically lexical deficits (Gow, 2012; Hickok & Poeppel, 2007). This implies that decoding of lexical neighbors by posterior STG reflects segmental representations (Gwilliams et al., 2022; Mesgarani et al., 2014) that are enhanced by resonance with word level representations in posterior MTG. These representations may be further stabilized by reciprocal connections over a larger network of wordform-informed representations of articulation in inferior postcentral gyrus (R-postCG_5_) and representations integrating sensory, motor and linguistic representations in R-ITG_3_, a portion of anterior inferior temporal cortex with MNI coordinates consistent with the anterior temporal pole (Patterson et al., 2007; Small et al., 1997). These effective connectivity patterns demonstrate the propagation of wordform representation through a distributed network of regions involved in spoken word recognition.

Taken together, our results provide evidence of a functional neural representation of wordform in the right temporal cortex, with contributions from left STG. The convergence of decoding and effective connectivity results with an existing experimental literature implicating this region in form-based lexical processes satisfies the requirements for demonstrating representation identified by Dennett (1987) and Kriegeskorte and Diedrichsen (2019). This approach provides a potential template for future experimental exploration of neural representation. While the current results provide a limited window into the content of representations of wordform, the finding that representations evoked by phonologically similar words involve sufficiently similar patterns of neural activity to support transfer decoding demonstrates that wordform representations systematically encode aspects of phonological structure rather than simply individuating words or the mapping to syntactic or semantic information. We suggest that future work exploring the content of these representations may shed light on the role that lexical representation plays in listeners’ ability to discriminate phonemic minimal pairs, while still being able to recognize words despite systematic phonemic disruption resulting from phenomena including lawful phonological processes (e.g., assimilation), reduced speech, or dialectal variation.

## Supplementary Information

Below is the link to the electronic supplementary material.

Supplementary file1 (DOCX 595 KB)

## Data Availability

The authors confirm that the data supporting the findings of this study are available within the article [and/or] its supplementary materials through the Harvard Dataverse (Gow, David, 2024, "Replication Data for Gang EffectsStudy", 10.7910/DVN/8FOV1J, Harvard Dataverse, V1). Code and documentation for our Granger Processing Stream (GPS) is available at https://www.nmr.mgh.harvard.edu/software/gps. GPS relies on Freesurfersoftware for MRI analysis and automatic cortical parcellation (https://surfer.nmr.mgh.harvard.edu/) and MNE software for reconstruction of minimum norm estimates of source activity based on combined MRI, MEG and EEG data (https://pypi.org/project/mne/). Support vector machine code can be found at (www.csie.ntu.edu.tw/~cjlin/libsvm).
