# Supplementary material for: Neural representation of phonological wordform in temporal cortex
Source: Psychon Bull Rev. 2024 Apr 30;31(6):2659–71. doi: 10.3758/s13423-024-02511-6 (PMC11680662; doi:10.3758/s13423-024-02511-6)
Supplement: Supplementary file 1 — Supplementary file1 (DOCX 595 KB) [file 13423_2024_2511_MOESM1_ESM.docx]

Supplementary Material

**Table S1.** Phonological neighborhood stimuli used in this study.

| **Hub Words** |  | **Word Neighbors** |  |  | **Nonword Neighbors** |  |
| --- | --- | --- | --- | --- | --- | --- |
| pig | big | peg | pick | tig | poog | pid |
| toad | code | tad | tote | dode | taid | toab |
| cab | tab | cob | cap | pab | keb | kag |
| bike | pike | beak | bite | gike | boke | bipe |
| dupe | goop | dope | duke | toop | dup | doot |
| gut | cut | get | guck | dut | geet | gud |

**Table S2.** Regions of interests (ROIs) used in neural decoding and Granger causation analyses, as identified from averaged task-related activation. average activation across participants within each region.

| **Label** | | **Location** | **MNI Coordinates** | | |
| --- | --- | --- | --- | --- | --- |
| **Left Hemisphere** | |  | **X** | **Y** | **Z** |
| Temporal | L-STG_1_ | Superior Temporal Gyrus | -67 | -22 | -2 |
|  | L-STG_2_ | Superior Temporal Gyrus | -55 | 9 | -9 |
|  | L-MTG_1_ | Middle Temporal Gyrus | -62 | -59 | 7 |
|  | L-MTG_2_ | Middle Temporal Gyrus | -65 | -14 | -18 |
|  | L-TPol_1_ | Temporal Pole | -29 | 17 | -35 |
|  | L-ITG_1_ | Inferior Temporal Gyrus | -54 | -40 | -31 |
|  | L-ITG_2_ | Inferior Temporal Gyrus | -51 | -12 | -41 |
| Parietal | L-SPC_1_ | Superior Parietal Cortex | -16 | -49 | 75 |
|  | L-SMG_1_ | Supramarginal Gyrus | -64 | -28 | 31 |
|  | L-postCG_1_ | Posterior Central Gyrus | -63 | -6 | 11 |
| Frontal | L-SFG_1_ | Superior Frontal Gyrus | -14 | -3 | 72 |
|  | L-cMFG_1_ | Caudal Middle Frontal Gyrus | -39 | 20 | 52 |
|  | L-ParsTri_1_ | Pars Triangularis | -56 | 25 | 7 |
|  | L-ParsOrb_1_ | Pars Orbitalis | -49 | 33 | -16 |
| Medial | L-CC_1_  L-ParaHip_1_ | Corpus Callosum  Parahippocampal Cortex | 1  -18 | 5  -21 | 22  -27 |
| **Right Hemisphere** | | | | | |
| Temporal | R-STG_1_ | Superior Temporal Gyrus | 69 | -27 | 7 |
|  | R-STG_2_ | Superior Temporal Gyrus | 67 | -11 | 1 |
|  | R-STG_3_ | Superior Temporal Gyrus | 55 | 7 | -6 |
|  | R-MTG_1_ | Middle Temporal Gyrus | 69 | -42 | -4 |
|  | R-MTG_2_ | Middle Temporal Gyrus | 67 | -24 | -10 |
|  | R-MTG_3_ | Middle Temporal Gyrus | 63 | -12 | -14 |
|  | R-MTG_4_ | Middle Temporal Gyrus | 53 | 1 | -26 |
|  | R-ITG_1_ | Inferior Temporal Gyrus | 55 | -58 | -19 |
|  | R-ITG_2_ | Inferior Temporal Gyrus | 55 | -36 | -30 |
|  | R-ITG_3_ | Inferior Temporal Gyrus | 53 | -20 | -37 |
| Parietal | R-AG_1_ | Angular Gyrus | 47 | -60 | 48 |
|  | R-postCG_1_ | Postcentral Gyrus | 16 | -32 | 77 |
|  | R-postCG_2_ | Postcentral Gyrus | 45 | -18 | 57 |
|  | R-postCG_3_ | Postcentral Gyrus | 56 | -10 | 38 |
|  | R-postCG_4_ | Postcentral Gyrus | 63 | -16 | 27 |
|  | R-postCG_5_ | Postcentral Gyrus | 64 | -6 | 17 |
|  | R-preCG_1_ | Precentral Gyrus | 47 | 4 | 42 |
|  | R-preCG_2_ | Precentral Gyrus | 56 | 7 | 16 |
| Frontal | R-SFG_1_ | Superior Frontal Gyrus | 7 | 12 | 68 |
|  | R-SFG_2_ | Superior Frontal Gyrus | 18 | 33 | 55 |
|  | R-SFG_3_ | Superior Frontal Gyrus | 8 | 62 | 28 |
|  | R-ParsOrb_1_ | Pars Orbitalis | 51 | 32 | -13 |
| Occipital | R-LOC_1_ | Lateral Occipital Cortex | 25 | -98 | -16 |


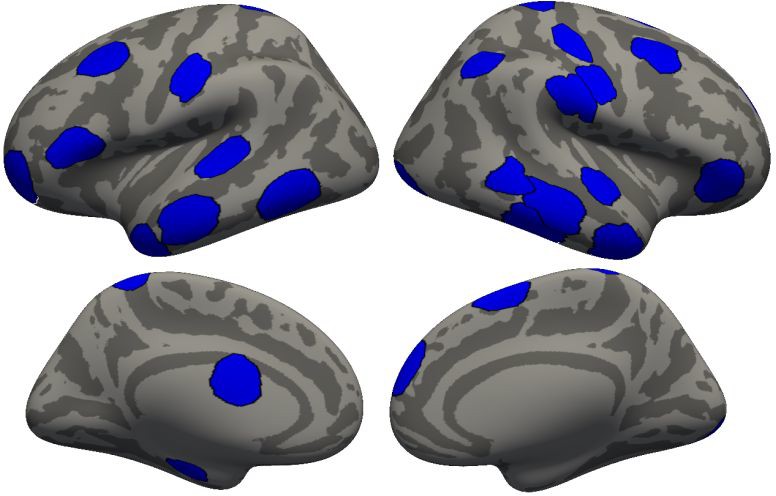


**Figure S1** Example ROI set generated using parameters in previous applications of the Granger Processing Stream analysis software as detailed in Gow and Nied (2014). A total of 26 ROIs are generated in this set, as compared to the 39 in the manuscript (compare Figure 1).


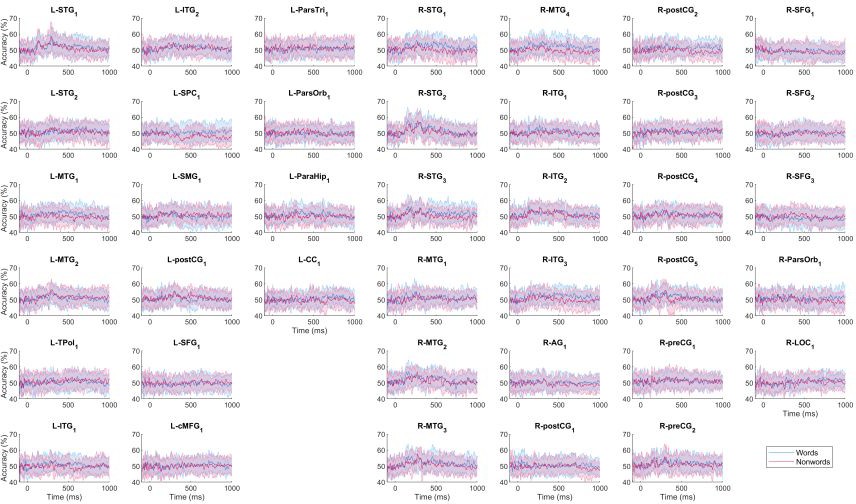


**Figure S2.** Transfer decoding accuracy timecourses in lexicality effects analyses. Grand average transfer decoding timecourses by ROI after training with word neighbors (blue) or nonword neighbors (red). Shaded area represents one standard deviation


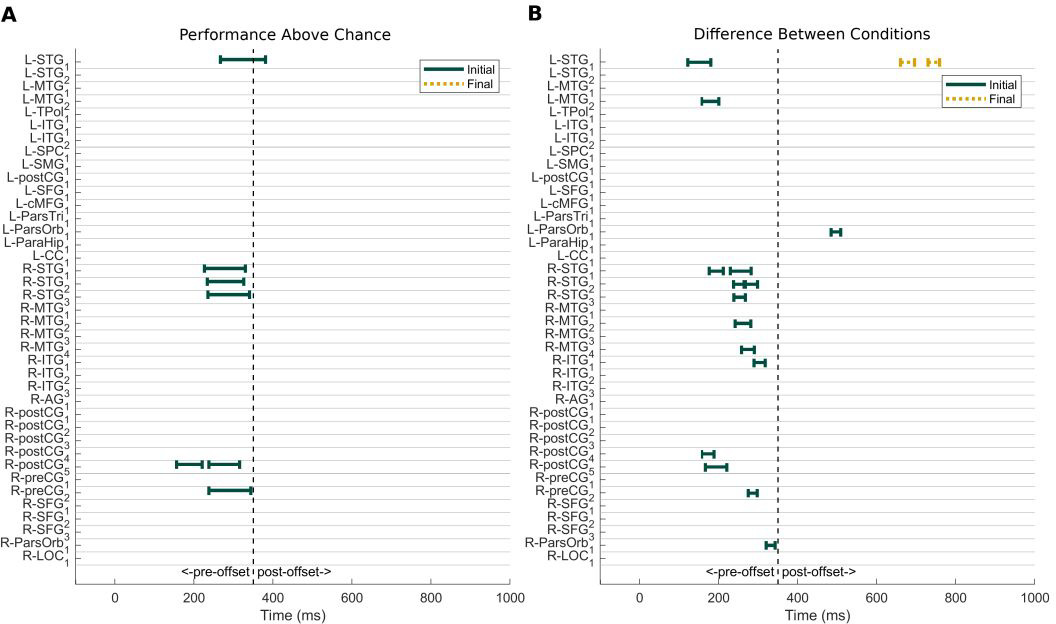


**Figure S3.** Transfer decoding results when trained with shared initial or final consonant neighbors. A) Clusters of significant above chance (Bonferroni-corrected alpha = 0.05) transfer decoding accuracy for initial-only (green solid) or final-only (yellow dotted; none observed) conditions. B) Clusters of transfer decoding accuracy which is above chance and significantly differs between training conditions. Better transfer decoding for initial-only condition indicated by green solid bars, and better transfer decoding for final-only conditions indicated by yellow dotted bars. The vertical dotted line indicates the offset of the auditory stimulus.


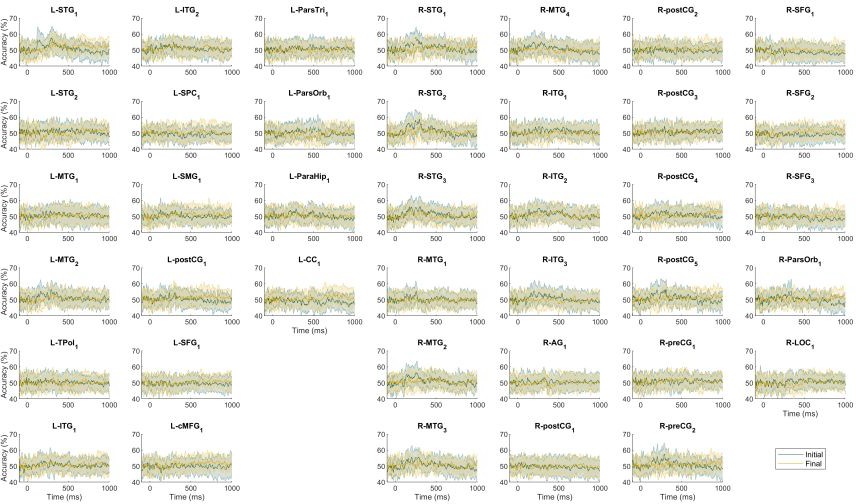


**Figure S4** Transfer decoding accuracy timecourses for the positional effects analyses. Grand average transfer decoding timecourses by ROI after training with neighbors based on a shared initial consonant 27 (green) or shared final consonant (yellow). Shaded area represents one standard deviation.

References

Gow, D., & Nied, A. (2014). Rules from words: Phonotactic biases in speech perception. PloS One, 9(1), 1-12. https://doi.org/10.1371/journal.pone.0086212
